# Data-efficient human walking speed intent identification

**DOI:** 10.1017/wtc.2023.15

**Published:** 2023-07-03

**Authors:** Taylor M. Higgins, Kaitlyn J. Bresingham, James P. Schmiedeler, Patrick M. Wensing

**Affiliations:** Department of Mechanical Engineering, Florida A&M - Florida State University, Tallahassee, FL, USA

**Keywords:** Real-time models, Human-robot interaction, Biomechanics

## Abstract

The ability to accurately identify human gait intent is a challenge relevant to the success of many applications in robotics, including, but not limited to, assistive devices. Most existing intent identification approaches, however, are either sensor-specific or use a pattern-recognition approach that requires large amounts of training data. This paper introduces a real-time walking speed intent identification algorithm based on the Mahalanobis distance that requires minimal training data. This data efficiency is enabled by making the simplifying assumption that each time step of walking data is independent of all other time steps. The accuracy of the algorithm was analyzed through human-subject experiments that were conducted using controlled walking speed changes on a treadmill. Experimental results confirm that the model used for intent identification converges quickly (within 5 min of training data). On average, the algorithm successfully detected the change in desired walking speed within one gait cycle and had a maximum of 87% accuracy at responding with the correct intent category of speed up, slow down, or no change. The findings also show that the accuracy of the algorithm improves with the magnitude of the speed change, while speed increases were more easily detected than speed decreases.

## 1. Introduction

Identifying human gait intent is an emerging challenge for robotics applications that involve planning for, and integrating with human locomotion (Babič et al., 2021). Autonomous mobile robots may navigate around moving humans (Aoude et al., 2013), whereas robotic lower-limb prostheses (Wentink et al., 2013; Yuan et al., 2022) and exoskeletons (Jung et al., 2012; Zhang et al., 2022b) support stable, efficient gait in coordination with the user. These applications all require the ability to reason about what the human aims to accomplish (Malmi, 2013; Abbink et al., 2018; Chauhan et al., 2019; Khalin et al., 2021; Kalinowska et al., 2023). Robots assisting locomotion rely on a variety of sensors to glean information related to the human’s intentions. Electromyography (EMG) is arguably the most popular sensing modality for intent identification in this domain (Hargrove et al., 2015; Chen et al., 2017). EMG-sensed muscle potentials map to desired joint torques by various means, one of which is applying robotic joint torques proportional to the EMG signal (Young et al., 2017; Fleming et al., 2021). These methods succeed when the user has sufficient control over the sensed muscles, which can be compromised by neuromuscular injury like stroke or spinal cord injury.

**Figure 1.**
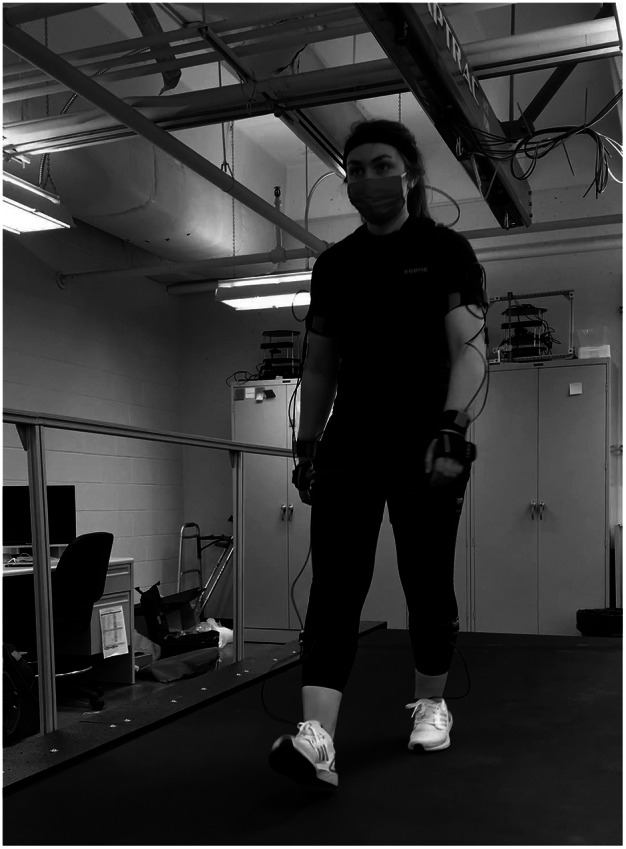
Subject wearing XSens inertial motion capture suit, making changes of intended walking speed on large research treadmill.

Electroencephalography (EEG) is an alternative that measures electrical potentials across the user’s scalp. Since these movement-related cortical potentials exist even when a user simply thinks about moving (Bi et al., 2013; Wang et al., 2018; Vinoj et al., 2019), EEG does not require true motor control over the remaining physiology. EEG signals have been used mainly to detect only high-level gait intentions like starting/stopping (He et al., 2018) and have not provided detailed information about desired individual joint motions. While more invasive forms of brain sensing (intercranial EEG) could potentially identify detailed joint-level intentions (Gilja et al., 2011), this level of invasiveness is not appropriate for every person with gait deficits who may benefit from using an assistive device. In contrast to both EMG and EEG, which sense the user directly, intent information can be inferred from the physical connections between the human and robot without direct human measurements. Ground reaction force sensors, torque sensors, motor encoders, motor current draw, and inertial measurement units (IMUs, such as in the motion capture suit in Figure 1) can determine progression through the gait cycle, which helps infer appropriate robot action (Pacini Panebianco et al., 2018; Gambon et al., 2019; Elery et al., 2020; Lazzaroni et al., 2020; Naseri et al., 2020; Liang et al., 2021; Tan et al., 2021).

Many of the intent identification strategies described above are sensor-specific, so disparities in sensing resources across commercially available devices have made comparisons difficult. In contrast, pattern recognition approaches depend less on *which* sensors are used and more on the *information content* available from the data. Pattern recognition is most often pursued with long short-term memory networks (Romero-Hernandez et al., 2019; Kim et al., 2022), convolutional neural networks (Casale et al., 2011; Zhang et al., 2022a), or other architectures that have proven useful for the gait intent recognition or prediction tasks (Fang et al., 2020). While these strategies reliably predict human joint movements, they typically require massive amounts of training data. For instance, Fang et al. (2020) used 598 min of walking data from seven IMUs attached to the lower limbs. Eighty percent of the data were used to train their gait neural network that both classified the gait as standing, walking, or running and predicted the velocities and accelerations of each limb. The resulting network did fairly well at generalizing from a model trained with one user and tested on another without additional model training (mean squared error of 0.0144 increased to 0.0277 for prediction, and accuracy decreased from 100% to 98.04% for classification - units for error are unclear because they incorporate errors across measurements with different units). Therefore, 589 min of training data would not be required for each device user, which is sometimes referred to as a “user-independent” solution (Kang et al., 2022). All subjects, however, were able-bodied. Subjects with neuromuscular disorders would typically exhibit more highly individualized movement patterns, so they would likely need to provide a substantial amount of their own training data to achieve similar results. It is even more onerous for this population to supply such training data due to time constraints during therapy, fatigue, and changes to their condition over time.

This paper presents a data-efficient, real-time algorithm for identifying when the human wishes to speed up (SU), slow down (SD), or make no change (NC) in walking speed. The algorithm leverages the cyclic structure of human walking by assuming that any sensor measurements should be roughly the same at the same time step of the gait cycle each time the cycle is repeated, as long as the individual is walking with constant speed intent. As such, each time step of the cycle is modeled as a random variable that follows a multivariate Gaussian distribution. By assuming that the distribution for each time step is independent of all other time steps, the model converges with just 5 min of constant-speed-intent training data for healthy subjects.

This data-driven approach works for multiple sensor suites as opposed to being device-specific. In fact, preliminary work employed motor encoders and motor current data from the EksoGT exoskeleton (Gambon et al., 2020), and the experiment herein employed an inertial motion capture suit (Xsens). For this application, the algorithm monitors the joint angles of the human subjects as they walk in the suit and identifies when the joint angle trajectories deviate from their expected pattern for constant walking speed. Because the algorithm relies on changes to the joint angle trajectories, not on the actual walking velocity, it is possible to identify trajectory changes that are indicative of an intention to change walking speed even if the changed trajectories have not achieved the newly desired walking speed. This is especially important when the user is incapable of achieving the desired speed change independently, but the assistive device may help them to do so if it has identified the intention. This work deals exclusively with identifying the human’s gait intentions and does *not* suggest how a robotic device might respond. In fact, the Xsens is not a robotic device, nor does it provide any walking assistance. The use of the Xsens highlights the utility of the approach for implementation with devices as disparate as exoskeletons, powered orthoses, and powered prostheses. This paper first introduces the algorithm generally, such that it could be applied to any sensors, and then assesses its accuracy and time delay with XSens motion capture data in an Institutional Review Board (IRB)-approved human subject study. Results from this study are also reported in Chapter 5 of Higgins (2022).

## 2. Methods

### 2.1. Mahalanobis distance intent algorithm

At a high level, the first step of the algorithm involves training a gait model on a set of data that is representative of the sensor measurements expected for constant walking speed intent. Once trained, incoming data can be compared to the gait model to determine if the new data are different enough from the model to be considered indicative of a change of intended speed.

From a more detailed perspective, begin by considering the vector of 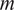 sensor measurement values as a random variable 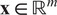 . When the user is walking with constant speed, 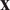 is assumed to follow a multivariate Gaussian distribution whose mean and covariance depend on the time step within the gait cycle according to the following equation:
(1)

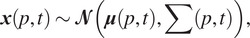

where 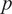 represents the gait phase

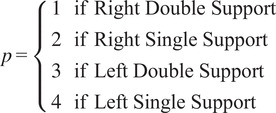




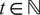
 (the natural numbers) represents the number of time steps since that phase began, 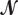 symbolizes a Gaussian distribution, 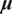 is the mean vector of the distribution, and 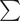 is the covariance matrix of the distribution. Right/Left Double Support denotes the double-support phase in which the right/left leg is leading, and Right/Left Single Support indicates that only the right/left leg is in contact with the ground. For example, the distribution of 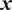 at the twentieth time step within the Left Double Support phase is described by 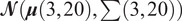 .

The model in Eq. (1) implies that 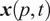 comes from a cyclostationary random process (Zakaria et al., 2013)––the distribution at each combination of 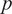 and 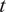 is stationary while the user walks with constant walking speed intent. This model also ignores the correlation between the Gaussian distributions at different time steps. While time dependency could be considered (e.g., with a traditional Gaussian Process model), this assumption reduces the complexity of model training and ultimately reduces the amount of data required to train the model to convergence. Each mean and covariance can be estimated with the sample mean and covariance at each time step for a set of training observations, which is equivalent to finding the maximum likelihood Gaussian distribution for the data at each time step (Eliason, 2011). To add data to the model one observation at a time, the mean and covariance estimates after the 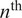 training observation are computed recursively via
(2)

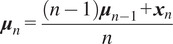



(3)

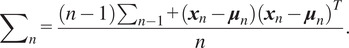

**Figure 2.**
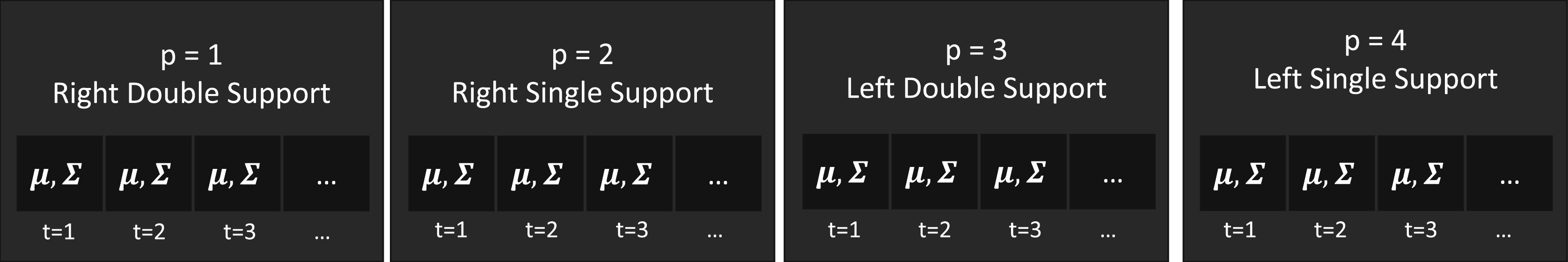
Model structure. Each time step in each phase includes measurement mean 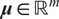 and covariance 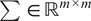 , where 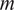 is the number of sensor measures being used.

The training data to build this model are collected as the user walks at a constant speed and organized in the structure of Figure 2 by determining first to which of the four gait phases the data belong 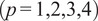 and then how many time steps have elapsed since the start of that phase ( 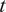 ). Preliminary results showed that dividing the gait cycle into these four phases resulted in greater accuracy and responsiveness than considering the whole gait cycle without phase divisions. The most appropriate method to determine gait phase in real time is likely based on the available sensors. For example, the EksoGT exoskeleton has built-in foot contact sensors for direct phase detection (Kalinowska et al., 2019), whereas motor encoders or IMUs allow for inference of phase based solely on the leg kinematics (Grimmer et al., 2019).

**Figure 3.**
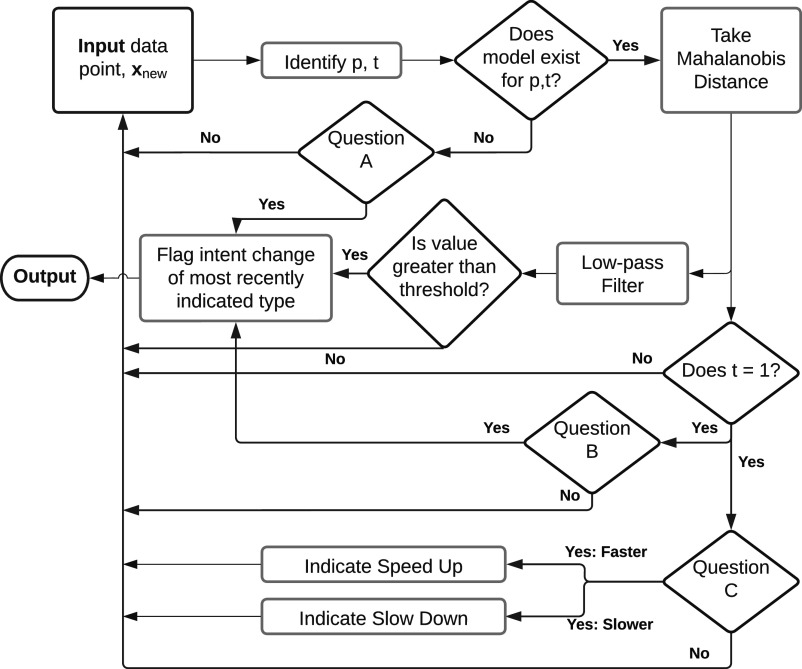
Real-time walking speed intent identification flow chart. **Question A:** Is difference between current 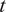 & number of time steps in model for this phase greater than one standard deviation of number of time steps for this phase from training set? **Question B:** Is difference in number of time steps between previous phase and model for that phase greater than one standard deviation of number of time steps for this phase from training set? **Question C:** Is this the start of a new gait cycle? If so, was the most recently completed cycle faster or slower than the mean gait cycle from the training set?

The real-time intent identification algorithm shown in Figure 3 begins with a single new measurement data point 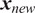 . The current gait phase 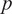 and time step 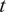 for 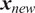 are determined just as in model building. If this 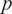 and 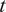 combination exists in the model, the current data point 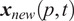 is compared to that part of the model by assessing the Mahalanobis distance (MD) between it and the Gaussian distribution (Zelterman, 2015),
(4)

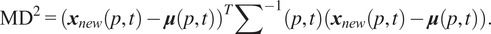

The MD is a metric for assessing how far a single point is from the mean of a Gaussian distribution, by scaling the Euclidean distance between the new point and the mean of the distribution by the uncertainty (variance/covariance) in each dimension. The squared MDs of samples from a normal distribution follow a 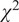 distribution with the same number of degrees of freedom. Therefore, evaluation of the 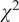 cumulative distribution function (CDF) for a squared MD gives the probability that any other sample from the given distribution will have a smaller MD than the one being evaluated. Essentially, it provides the likelihood that another sample would be closer to the mean of that distribution than the point being evaluated. At one extreme, a 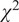 CDF value of one implies that the given data point is almost certainly not a sample from the model distribution, indicating changed walking speed intent. Likewise, a 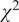 CDF value of zero implies that the data point is the exact mean value of the model distribution, indicating that speed intent has not changed. Therefore, the 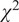 CDF helps identify the MD threshold above which a walking speed intent change is indicated. For instance, if an MD threshold represents a 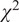 CDF value of 0.99, then incoming data must be farther from the mean than 99% of data from the modeled distribution in order to indicate a walking speed intent change. In real time, the MD signal should be low-pass filtered before comparison to any threshold, as noise in the raw data signal can cause toggling between indications of a speed intent change and NC.

Given a new measurement 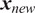 , the first task is to determine whether or not a walking speed intent change has occurred (a yes/no question). When a gait phase observation has the same number of time steps as the modeled training data, there is always a Gaussian distribution model available with which to take the MD. In this case, if the filtered MD value rises above the threshold value at any point, a walking speed intent change is flagged. When there is a mismatch in time steps between the incoming observation and the gait model, however, there may not always be a Gaussian distribution to which to compare. For instance, when a gait phase observation has more time steps than the gait model, there is no Gaussian distribution with which to take the MD. If the timing mismatch is greater than the standard deviation in phase timing from the training set, a speed intent change is flagged. Thus, even without an MD signal for these data points, the algorithm still leverages the timing offset as a possible indication of a speed intent change. When a gait phase observation has fewer time steps than the gait model, there is always a Gaussian distribution with which to take the MD, but some time steps of the model are skipped because the gait has already proceeded to the next phase. In this situation, if the timing mismatch is greater than the standard deviation in phase timing from the training set, a speed intent change is flagged.

If there has been a change in intended walking speed, the next task is to determine which *type* it was (SU or SD). As a distance metric, the MD is strictly positive, so it cannot differentiate the type of intent change. The type of change can, however, be determined by speed mismatches between the incoming gait cycle observations and the expected walking speed from the constant-intent training data. Regardless of the size of the mismatch, the algorithm always tracks whether the most recently completed gait cycle was completed with a higher/lower speed than expected from the training set. If and only if a speed intent change is triggered by a mechanism described in the preceding paragraph, the type is assigned to the type indicated by the most recent speed mismatch. For instance, if a subject is walking, the algorithm has identified an intent change, and the most recently completed gait cycle was slower than expected from the training set, then the change will be classified as an SD. This design is inspired by (Gambon et al., 2020), which showed that when an intent change has been made, there are changes both to the values of the onboard sensor measures and to the speed of the gait cycle. Preliminary data showed that the variability of speeds within individual gait phases, even during walking at constant speed intent, was greater than the variability in speed across the whole gait cycle, which is why the full cycle duration is used here to indicate the type of intent change.

To summarize, for each new data point there are three main tasks: (1) determine the appropriate Gaussian distribution with which to take the MD; (2) determine if a walking speed intent change has occurred (yes/no); and (3) if there has been a walking speed intent change, determine which type it represents (SU/SD).

### 2.2. Human subject experiment

Fifteen able-bodied human subjects gave their informed consent and participated in this study approved by the IRB of the University of Notre Dame (Protocol no. 19-10-5589). Subjects donned the XSens inertial motion capture suit (full-body configuration with 16 IMUs) and performed the recommended calibration sequence (a short walk, about-face, and return to the starting position) in a hallway, well away from magnetic disturbances, such as from the large treadmill shown in Figure 1 on which experiments were performed. After calibration, subjects walked on the treadmill for 5 min at a constant 1.4 m/s (the average healthy adult human’s preferred walking speed (PWS; Malatesta et al., 2017) to provide the model training data (Eqs. 2 and 3). This training data was not used to test the algorithm’s performance.

**Figure 4.**
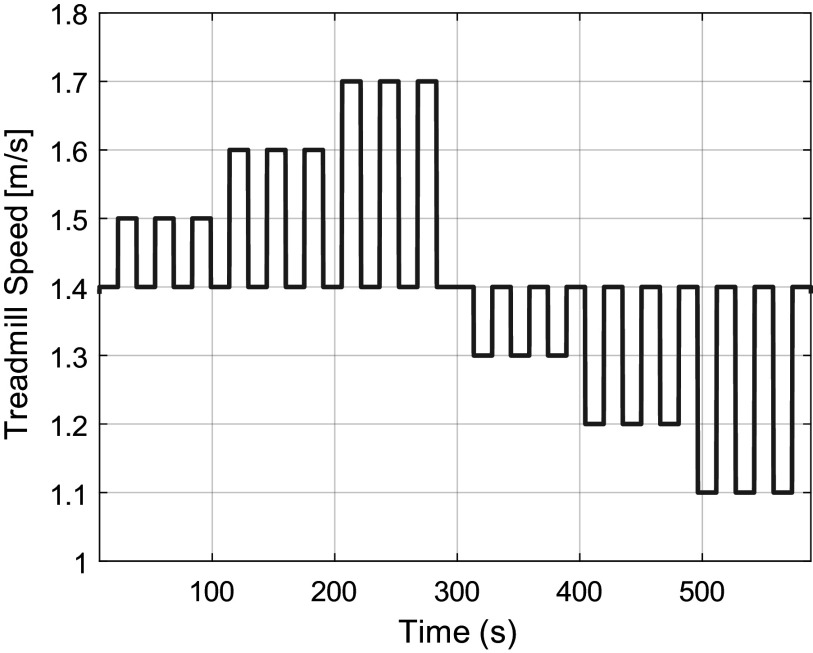
The speed trajectory of the treadmill for each testing set. The treadmill completes three small (0.1 m/s), three medium (0.2 m/s), and three large (0.3 m/s) speed increases and then speed decreases.

Next, each subject was instructed to stay within a small area on the treadmill, marked by two elastic bands strung across the handrails, while the treadmill imposed a set of speed changes, the schedule of which was explained to them before beginning. The speed first ramped up from 0 m/s to 1.4 m/s over several seconds, then nine speed-up trials and nine slow-down trials were performed, as illustrated in Figure 4. Each set of nine trials consisted of three small (0.1 m/s), three medium (0.2 m/s), and three large (0.3 m/s) speed changes. For each, the treadmill spent 15 s at the baseline of 1.4 m/s, accelerated/decelerated at 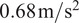 (average acceleration for daily pedestrian flow (Teknomo, 2002) until reaching the new speed, remained at the new speed for 15 s, and then returned to 1.4 m/s.

While the size and type of speed change of each trial could have been randomized instead of occurring in a fixed order, pilot study subjects reported unease about the possibility of being startled by the treadmill making random speed changes. As this study seeks to recreate speed changes that subjects might perform in their activities of daily living, the fixed schedule was determined to recreate the fact that humans generally know when they are about to change speeds and are not startled by their own actions.

### 2.3. Real-time processing

**Figure 5.**
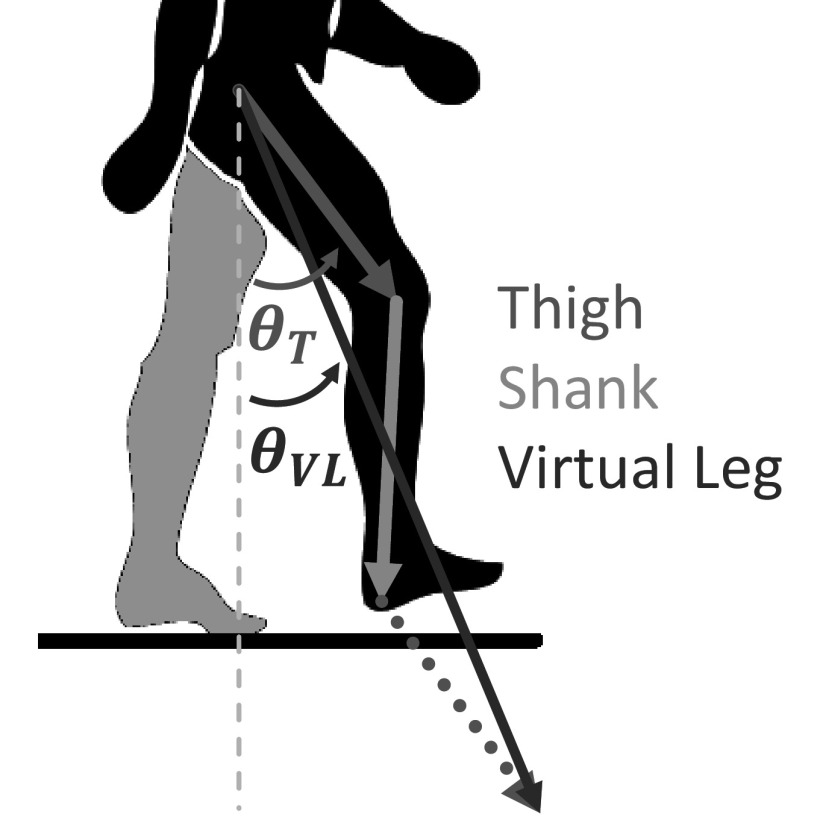
Illustration of the virtual leg vector (blue), which is the result of adding two times the thigh vector (orange) and the shank vector (green). The angle that this virtual leg makes with the vertical is monitored to determine when a leg is in swing (when this angle increases) and in stance (when this angle decreases).

The user’s intentions were analyzed in real time by first determining the current gait phase for each data point. Each leg was determined to be in stance or swing based on the angle with the vertical made by a virtual extended leg vector. This virtual leg vector is defined by adding two times the vector that connects the hip to the knee, plus the vector connecting the knee to the heel (Figure 5) (Grimmer et al., 2019). The virtual leg vector does not track the true biological movement of a specific landmark on the body, but the angle that it makes with the vertical increases/decreases monotonically during swing/stance. With only one leg in stance, Right/Left Single Support 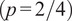 was assigned based on the stance leg. With both legs in stance, Right/Left Double Support ( 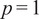 or 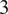 ) was assigned based on the leading leg. For each model phase, 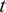 was tracked by finding the nearest 0.01 s (each red block in Figure 2) since the phase’s beginning and reset to 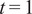 when the incoming data switched to a new phase. With 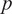 and 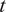 determined, data were compared to the appropriate Gaussian distribution by taking the MD. The squared MD signal was filtered with a first-order low-pass Butterworth filter with a cut-off frequency of roughly 1.2 Hz. The squared MD threshold was set at 13.5, which corresponds to a 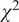 CDF value of 0.99. The exact procedure for selecting this threshold value is discussed in the following section.

### 2.4. Data analysis

It is important that the performance of the algorithm be contextualized with respect to the convergence of the constant-speed gait model. For instance, if the gait model had not converged, more training data would likely be necessary to reach the algorithm’s maximum performance. If the model has converged, however, the performance of the algorithm is likely as high as can be expected. For the algorithm to be considered data-efficient, it must rapidly converge to a stable model of the constant-speed gait and perform accurately thereafter.

The MDs of samples from a Gaussian distribution follow a 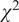 distribution and by the law of large numbers, as the sample size grows, the mean of the samples will converge to the mean of the true underlying distribution. Thus, as the amount of training data used to train the constant-speed gait model increases, the average MD of the training data with respect to said model should converge to the expected value of the 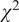 distribution, which is equal to the number of degrees of freedom of the distribution (Brereton, 2015) (four, in this case). In order to observe model convergence, the MDs of the 5-min training data set were taken with respect to gait models built with several subsets of the full training set (from 10–300 s worth of data). These MDs were calculated using the exact same algorithm as for the real-time implementation, including the low-pass filtering. The full vector of MDs, one for each training data point, was collected. Then the outliers were removed, which were defined as points that were beyond five times the inter-quartile range from the median. After outlier removal, the average of the MDs vector was taken. The average MD converging to four as the amount of data used to train the model increases is an indication that the model has converged to the anticipated underlying distribution.

The performance of the walking speed intent identification algorithm was then assessed by tabulating the number of instances of all possible correct and incorrect response types according to the confusion matrix template in Table. 1. The ground-truth labels were defined based on the speed of the treadmill. If the treadmill was running faster than, equal to, or less than 1.4 m/s, then the ground truth label was SU, NC, and SD, respectively. The confusion matrix tabulates the number of all possible correct and incorrect classifications by comparing the estimated intent output from the walking speed intent algorithm against the ground-truth intent. The first letter in each cell refers to the ground truth category (U for SU, D for SD, and N for NC), and the second letter to the category estimated by the algorithm (same possible letters). For instance, once the template is filled out, UU will represent the number of times an SU intent change was correctly identified, and DU the number of times an SD intent change was misidentified as an SU change. The number of True Positive (TP), True Negative (TN), False Positive (FP), and False Negative (FN) responses for each class were then calculated, as shown in Table 2.

**Table 1.** Confusion matrix that explains how every time step of output of the walking speed intent algorithm was compared with the ground-truth intent. The number of instances of each classification (UU, UD, UN, DU, DD, DN, NU, ND, and NN) is used with the equations in Table 2 to eventually calculate the performance metrics for each class via Eqs. (5)–(8)

	Estimated			
Ground truth		SU	SD	NC
	SU	UU	UD	UN
	SD	DU	DD	DN
	NC	NU	ND	NN

**Table 2.** Equations for calculating the number of True Positive (TP), True Negative (TP), False Positive (FP) and False Negative (FN) responses based on values from the confusion matrix

	Speed up	Slow down	No change
TP	UU	DD	NN
TN	DD + DN+…	UU + UN+…	UU + UD+…
	ND + NN	NU+NN	DU + DD
FP	DU + NU	UD + ND	UN + DN
FN	UD + UN	DU + DN	NU+ND

While more complex metrics exist for characterizing classifiers (Mortaz, 2020), the F1-Score (which depends on precision and recall), accuracy, and time delay are used here. Equations (5)–(7) are reproduced from Grandini et al. (2020)), where 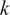 refers to a single class in the multi-class classification task (U for SU, D for SD, and N for NC):
(5)

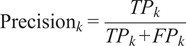



(6)

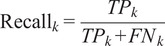



(7)

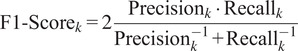



(8)

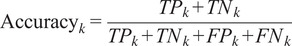

Time delay was defined from the instant the treadmill first began to change speed to the instant when the algorithm first identified the correct type of intended speed change.

**Figure 6.**
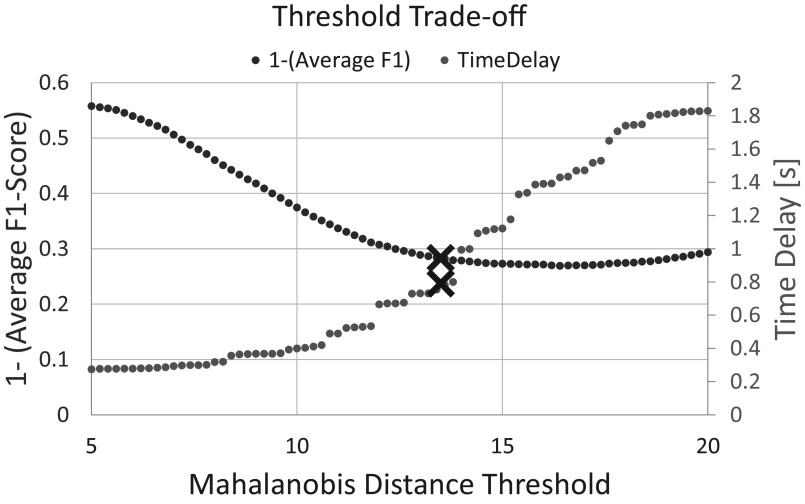
Illustration of the trade-off between the algorithm time delay and the average F1-score. Graphed is 1-Average F1-score on the left y-axis and the time delay on the right y-axis. Ideally, both values should be minimized. The values for the chosen Mahalanobis distance threshold of 13.5 are marked with a red 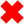.

In the preliminary analysis, it was observed that as the squared MD threshold was lowered, the time delay decreased, but the F1-scores also decreased as the algorithm produced more false-positive responses. Conversely, as the threshold was raised, the F1-score improved, but the time delay suffered. As shown in Figure 6 for a representative subject, an MD threshold of 13.5 showed a roughly equal trade-off between the F1-score and time delay.

A two-way analysis of variance (ANOVA) was used to test for statistically significant effects by the two independent variables, size of speed change, and category of intent classification. For significant effects associated with each dependent variable (precision, recall, F1-score, accuracy, and time delay), Tukey–Kramer posthoc tests determined which levels (small/medium/large for size and SU/SD/NC for category) were significantly different (Stolyarov et al., 2018). In other words, these statistical tests determined which sizes and types of intent change resulted in statistically significant algorithm performance. When results for recall were different than precision, further Tukey–Kramer tests were done on the TP/TN/FP/FN rates to reveal statistically significant differences in these algorithm responses. These true/false positive/negative rates are used to calculate all other performance metrics, so this secondary testing provides an even more detailed explanation of the algorithm’s performance. For all statistical tests, 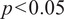 was considered significant. Following statistical analysis, the average results across all 15 subjects were calculated for each dependent variable.

## 3. Results

Results are shown first for the convergence of the constant speed model as the amount of training data grew (data from when subjects walked at a constant speed for 5 min). Then, the results are reported for model testing with the separate dataset from when subjects made predetermined speed changes. Results are organized by the model convergence analysis, the choice of the MD threshold value, and finally the overall algorithm performance.

### 3.1. Model convergence

**Figure 7.**
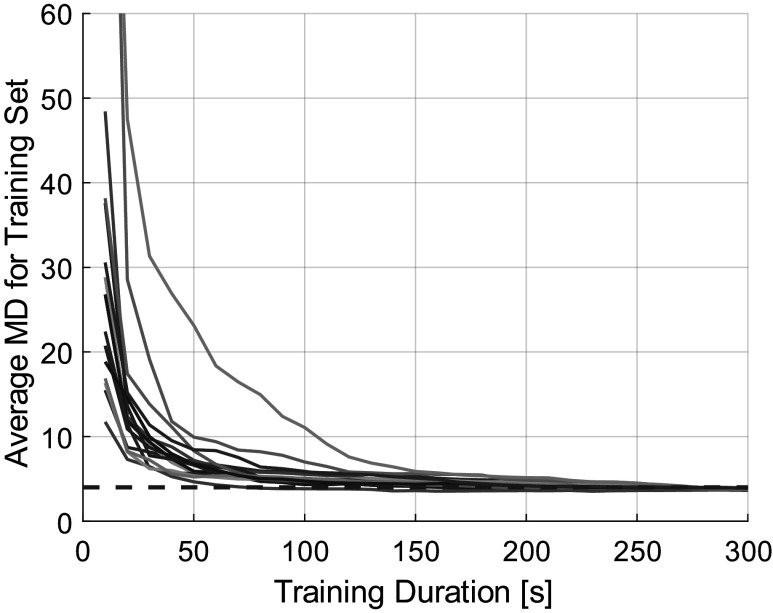
The average Mahalanobis distance of the training set with respect to gait models built on subsets of the training data ranging from 10 to 300 s of data. The red dashed line is at an average Mahalanobis distance of four, which is the theoretical mean for samples from a Gaussian distribution. Each curve represents a single subject.

The gait models can be seen to converge in Figure 7 as the amount of data used to train the gait model increased from 10 up to 300 s. Outliers represented only 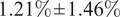 (mean 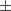 one standard deviation) of the data set (min 0.17%, max 12.94%) across the 15 subjects because the definition of an outlier was set so far from the median value (five times the inter-quartile range). It may seem erroneous that the standard deviation is larger than the mean, but this is a common occurrence, especially for data that is not normally distributed and may be skewed. Recall that the MDs of samples from a normal distribution follow a 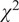 distribution, which is indeed a skewed distribution. The average MDs for the training set converged to within 10% of their lowest value (corresponding to the full 300-s-long training set) for each subject when the gait models were trained on 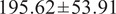 (max 270, min 70) s of constant-speed data. The average MDs for the training set across all subjects at the end of the 5-min training set was 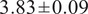 (max 3.93, min 3.63). This value is slightly less than the theoretically expected value of four because the outliers were removed.

### 3.2. Threshold analysis

Figure 6 illustrates the trade-off between the time delay and the average F1-score across the three classes (SU, SD, and NC) for various choices of the MD threshold. Ideally, the average F1-score should be maximized, and time delay should be minimized. Just for visual purposes, the value plotted is the average F1-score subtracted from 1, 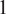 -(average F1-score), which is minimized if the average F1-score is maximized. In other words, the ideal behavior is when both curves in Figure 6 are minimized. Unfortunately, as the threshold is increased, 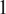 -(average F1-score) decreases and time delay increases, such that the MD threshold that produces the best time-delay produces an undesirable F1-score and vice-versa. The threshold chosen for the study was 13.5, which shows a roughly equal trade-off between F1-score and time delay.

### 3.3. Performance metrics

**Figure 8.**
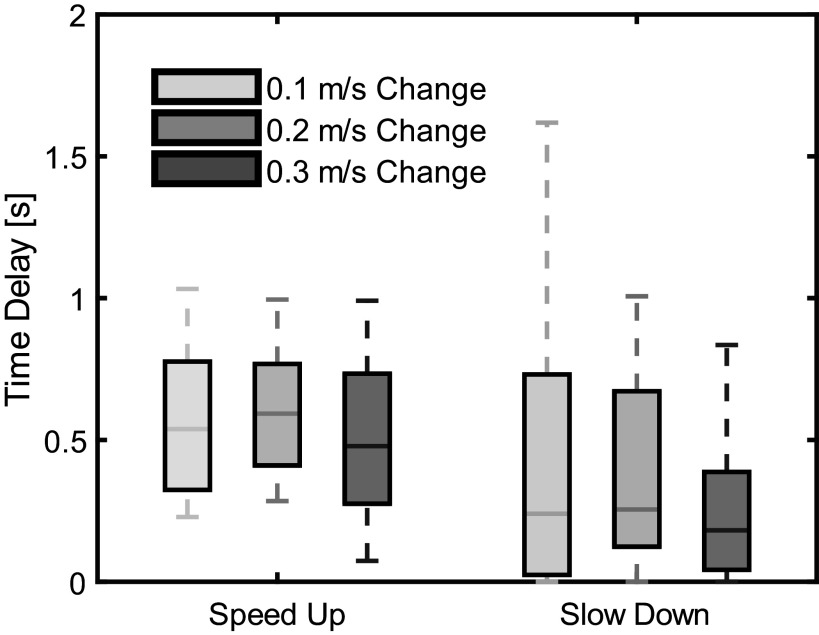
Algorithm performance for time delay across the three magnitudes of speed change and three categories of intent. The top and bottom of each box are the 75th and 25th sample percentile. Outliers, defined as a sample that was more than 1.5 times the interquartile range from the top or bottom of the box, are not indicated in this graph. Two outliers do exist, one for the 0.1 m/s change speed up (10.26 s) and one for 0.2 m/s change speed up (4.49 s). The top and bottom whiskers are the non-outlying maximum and minimum. The line within each box is the median of the sample.

**Figure 9.**
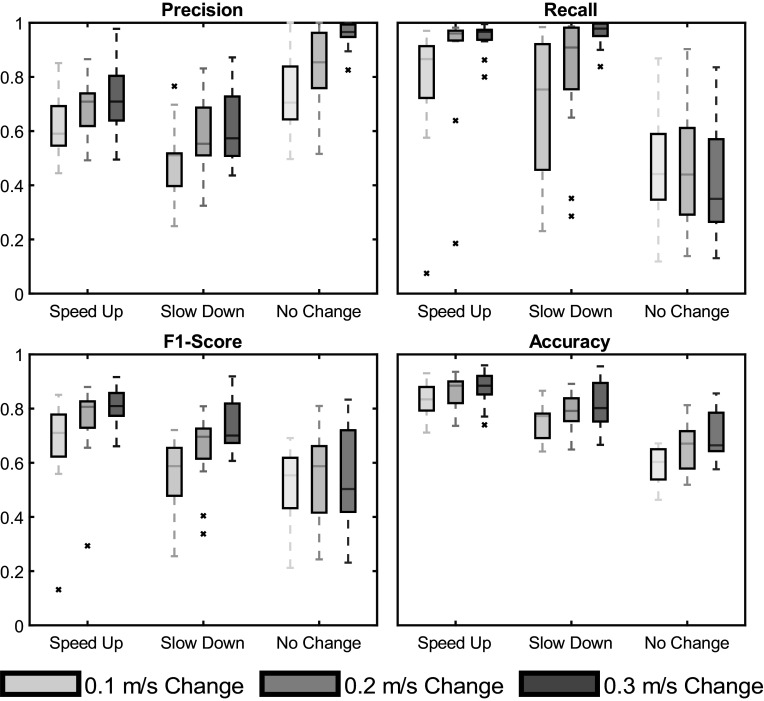
Algorithm performance for precision, recall, F1-score, and accuracy across the three magnitudes of speed change and three categories of intent. The top and bottom of each box are the 75th and 25th sample percentile. Each outlier, defined as a sample that was more than 1.5 times the interquartile range from the top or bottom of the box, are designated with an 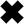. The top and bottom whiskers are the non-outlying maximum and minimum. The line within each box is the median of the sample.

The average performance metric values across all 15 subjects for each metric are reported in Figures 8 and 9. Time delay was best for the largest SD changes at 0.29 s and worst for the smallest SU changes at 1.19 s. Precision (increases with fewer false positives) had an average value across all conditions of 0.69 (max 0.96 for the NC condition of the largest speed changes and min 0.48 for the SD condition during the smallest speed changes). Recall (increases with fewer false negatives) had an average value across all conditions of 0.71 (max 0.97 for the SD condition of the largest speed changes and min 0.42 for the NC condition of the largest speed changes). F1-Score had an average value of 0.64 across all conditions (max 0.80 for the largest SU condition and min 0.52 for the NC condition for the smallest speed changes). Finally, Accuracy had an average of 0.76 across all conditions (max 0.87 for the largest SU conditions and min 0.59 for the NC condition during the smallest speed changes).

All performance metrics except recall for the NC condition improved as the magnitude of the speed change increased. The SU condition generally outperformed the SD condition (except for time delay), but the NC condition was the best category in precision (fewer false positives) and worst category for recall (greater false negatives). In general, this reluctance to assign the NC category means that the algorithm tended to over-indicate walking speed intent changes for an MD threshold of 13.5. Increasing the MD threshold would have mitigated this issue at the expense of the time delay.

The two-way ANOVA tested for main effects on the performance metrics by the size (small/medium/large) and category (SU/SD/NC) of intent classification. For all metrics except time delay, both the size and category of intent classification had a statistically significant effect, but the interaction effect was not significant. The size, category, and their interaction had no statistically significant effect on the time delay, although the category of change was nearly significant (*p* = 0.056).

For those metrics with statistically significant effects for size and/or category factors, the Tukey–Kramer posthoc tests showed which levels (0.1, 0.2, and 0.3 m/s change for size and SU, SD, and NC for category) within each factor were significantly different. The results for the post-hoc tests are shown in Table 3. Note that time delay does not appear in this table because neither factor had a significant effect for this metric in the two-way ANOVA. All three categories of intent were statistically significantly different from one another for all metrics except recall, where the SU versus SD comparison was not significant. Additional Tukey–Kramer tests revealed that SD had a significantly higher rate of FP responses (17.6% versus 11.4%) and a significantly lower rate of TN responses (57.5% versus 63.9%), but no other rates were significantly different than SU. For the size factor, only the small versus large speed change comparison was significantly different across all metrics.

**Table 3.** Results of the Tukey–Kramer post-hoc test for those factors found to be significant by the two-way ANOVA. Comparisons that were significant are indicated in bold and categorized into three levels of significance (*p* 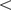 0.05, 0.01 or 0.001). For comparisons that were not significant, the *p*-value is provided

		Precision	Recall	F1-score	Accuracy
Category	SU vs. SD	***p* < 0.001**	*p* = 0.4625	***p* < 0.01**	***p* < 0.001**
	SU vs. NC	***p* < 0.001**	***p* < 0.001**	***p* < 0.001**	***p* < 0.001**
	SD vs. NC	***p* < 0.001**	***p* < 0.001**	***p* < 0.01**	***p* < 0.001**
Size	Small vs. medium	***p* < 0.01**	*p* = 0.1980	*p* = 0.0626	***p* < 0.05**
	Small vs. large	***p* < 0.001**	***p* < 0.01**	***p* < 0.001**	***p* < 0.001**
	Medium vs. large	*p* = 0.0721	*p* = 0.333	*p* = 0.2632	*p* = 0.1765

The bolded entries bring attention to the comparisons that were statistically significant.

## 4. Discussion

Discussion is divided between model convergence, category of classification, size of change, time delay, and future work.

### 4.1. Model convergence

For all subjects in this study, model convergence occurred well before the 5-min training set was complete. Future work may examine whether more extensive or different model training methodologies result in better algorithm performance, but with just 5 min of constant-speed training data, the algorithm reached an accuracy of 87% for the largest SU trials. For comparison, with similar intent identification algorithms using IMU measurements, Fang et al. (2020) required 589 min of training data and reached 100% accuracy, while Eyobu and Han (2018) required 150 min and reached 88.87% accuracy. It is worth noting that these two papers sought to classify standing/sitting/walking as the categories, while the present paper distinguishes different speed conditions within the walking regime, which is an inherently more difficult task because the classes are more similar to one another. Also worth noting is the fact that many papers on intent classification only report the accuracy value (Kim et al., 2018; Liu et al., 2019; Fang et al., 2020), rarely the precision, recall, or F1-score (Poliero et al., 2019). Accuracy is a good measure when TP and TN values are of the most importance and when the likelihood of each classification is balanced. The F1-score, however, does a better job of assigning importance to all types of responses, including FP and FN and handling classes of imbalanced probability (Hossin and Sulaiman, 2015). For speed intent recognition, it is of equal importance to have high TP and TN responses as well as low FP and FN responses, and there are no guarantees that the likelihoods of SU, SD, and NC will be balanced. Obviously, true responses are always good, but reducing FP and FN responses is also important. For example, an FP response may result in the assistive device speeding up when it is dangerous to do so, while an FN means that the device has not recognized the user’s intentions. Thus, the F1-score is a better discriminating metric for this application.

For an assistive device used by a single individual (e.g., a prosthetic limb), providing an extensive set of model training data when the device is first fitted may be acceptable. For other assistive device applications, it may be infeasible to collect such data. For example, most commercially available assistive exoskeletons are only available and FDA-approved for use in a physical therapy setting, where the device may have a new user as often as every hour. The physical therapy setting, therefore, is likely an area where the amount of time that can be spent training models is highly restricted. Ideally, model training would be done simultaneously with another preparatory stage of therapy. Prior to active therapy treatment, clinicians often have patients do a warm-up exercise of a duration highly dependent on the patient, but 10 min is common (Coons et al., 2017). Thus, the model training sequence presented herein can be completed during this “warm up” time, and would not disrupt therapy time that would otherwise be used for active treatment.

### 4.2. Category of classification

An increase in false-positive responses for SD trials indicates that even when subjects walked at 1.4 m/s (before the speed decrease), the gait was still different enough from the constant-speed model that intent change indications were triggered. Since the SD trials always happened after the SU trials, this phenomenon could be due to a drift in subjects’ baseline gait as more time passed since model training. This issue may be partially mitigated by increasing the threshold on the MD, but this would come at the cost of increasing time delay. Another way to mitigate drift may be to systematically add new baseline data to the model when the user is walking at the new constant speed, although it remains a topic for future investigation regarding determining when and how much data should be added.

Overall, the algorithm tended to under-assign the NC category. It is possible, however, that raising the MD threshold could result in a better F1-score average. Simply modifying the threshold value, however, cannot simultaneously increase the F1-score and decrease the time delay.

### 4.3. Size of change

It is not surprising that the largest magnitude of speed intent change resulted in the best algorithm performance. The larger the speed change, the more the joint angle trajectories would need to change, resulting in a larger MD (stronger intent signal) with respect to the constant-speed model.

While the algorithm was less accurate for the smallest speed intent changes, humans are less accurate at distinguishing speed changes of this size, too. For instance, the walking speed change that humans are able to perceive roughly 50% of the time is about 0.07 m/s (Liss et al., 2022). This means that if a gait assistance device were to misclassify the user’s desired walking speed by 0.1 m/s, the user may not even sense the discrepancy. From another perspective, large intent changes likely require more urgent responses than small ones. For instance, if the user needs to get out of the way of impending danger quickly, the present algorithm would indicate the intent change more reliably.

### 4.4. Time delay

To put the time delay results into perspective, the mean gait cycle duration across all subjects for the constant-speed training sets was 1.03 s. This means that for all conditions except the smallest SU, the algorithm generally classified the correct intent change within a single gait cycle. The time delays reported herein of 0.4–0.82 s are near the 0.3–0.6 s delays reported in the literature for similar intent recognition algorithms designed for prostheses (Varol et al., 2008; Khademi et al., 2019).

### 4.5. Future work

#### 4.5.1. Arbitrary speed detection

The algorithm described above is able to detect the desire to increase or decrease speed from a single baseline. Some preliminary work has been done to develop a modified algorithm that can detect the desire to walk at any arbitrary speed. The adaptation leverages the actual value of the MD as opposed to simply testing whether it is above or below a threshold. It is logical that the further the user’s desired speed is from the baseline, the larger the MD signal should get. For example, if the data indicates that the user would like to SU, but the MD is fairly small, the user’s desired speed can be inferred to be only slightly faster than the baseline (constant-speed) model. If, however, the data indicates that the user would like to SU, and the MD is quite large, the user’s desired speed can be inferred to be much faster than the baseline. To explore the viability of this approach, the authors collected some preliminary data from two pilot subjects. For each subject, joint angle trajectory signals were recorded using the XSens motion capture suit for 5 min of constant-speed walking at each of seven speeds (PWS, and then 0.1, 0.2, 0.3, and 0.4 m/s below PWS as well as 0.1 and 0.2 m/s above PWS). There were only two speeds recorded above PWS because the subjects were nearing the walk-to-run transition at these speeds. Subject-specific baseline gait models were trained on the data for each subject walking at their PWS. The data at other speeds were then compared to the baseline model by taking the MD following the exact same algorithm as described for the previous experiment.

**Figure 10.**
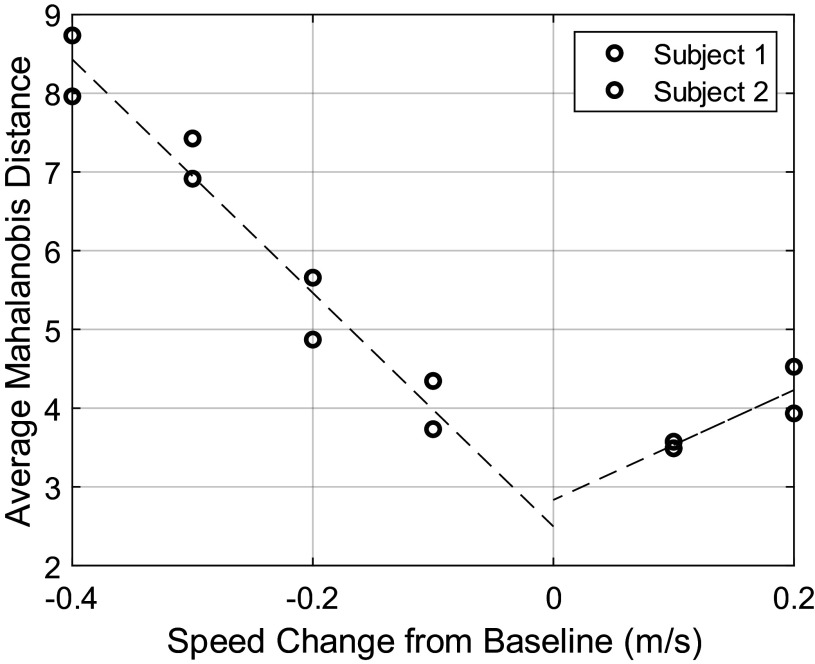
The average Mahalanobis distance compared to the baseline speed (subject’s preferred walking speed) as they walked at various other speeds. The dashed black line shows the line of best fit for the negative speed changes ( 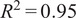 ) separately from the positive speed changes 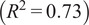 .

Figure 10 shows that there is a strong linear relationship between the average MD and the magnitude of the speed change away from baseline. Remarkably, the same linear trend appears to hold across two subjects who had very different PWSs (Subject 1 preferred 1.4 m/s while Subject 2 preferred 1.8 m/s). If this relationship continues to hold across subjects, it may be possible to train the model only at the user’s PWS and then use the linear trend to calculate any desired walking speed based on the value of the MD.

#### 4.5.2. Reacting to intent changes

While the work here focuses on identifying the user’s intentions to change speed and not on reacting to or incorporating those intentions into the device control scheme, these two tasks are likely to be highly coupled. For instance, an assistive device user’s tolerance for inaccurate intent identification probably depends heavily on the aggressiveness of the device’s response to the intent signal. If the device changes the control scheme only very slightly with each indicated intent change, the user may not even notice a few inaccurate intent change signals, but the device may be slow to respond to intentions. In this case, the user may not be able to detect the difference in accuracy between the data-efficient algorithm developed herein and the slightly more accurate but more data-heavy methods cited previously. If the device changes the control scheme aggressively with every indicated intent change, the user will likely be frustrated with every inaccurate response, despite the device being more responsive. Managing the trade-off between responsiveness and accuracy of the device that produces the most comfortable user experience is a topic of ongoing work Medina et al. (2015); Natarajan et al. (2022), although the optimal trade-off is likely to be user-specific. This tuning could be easily carried out with the proposed identifier by changing the MD threshold based on user preference.

The proposed algorithm may provide some hints as to how the user wishes to change the reference trajectory to better suit their needs. When an intent change is triggered for a specific time step, for instance, the location within the gait cycle of the indicated change is where the reference trajectory should be modified. The new data point that triggered the intent change signal is likely to contain information about how the user would like to modify the trajectory at this time step, but the interpretation of this modification will depend on the sensors and/or assistive device being used. For assistive devices like soft exosuits that do not restrict the user’s movements, for instance, the new joint trajectory can directly be taken as the user’s intended movement. For devices such as rigid exoskeletons that can restrict user movement, however, the forces that the user imparts on the device are likely to be more indicative of intentions than purely kinematic signals. If the user applies torques in resistance to the exoskeleton’s controller, this may indicate that the user wishes to modify the trajectory in the opposite direction of the exoskeleton torque. Future work should consider the location and direction of the change as well as the type of measurement (kinematic, torque, etc.) as clues to the user’s preferred movement and therefore to the ideal assistive device reaction.

#### 4.5.3. Other sensing modalities

When applied to a sensor suite with only kinematic sensors, as shown herein for the XSens motion capture suit, the user must be able to kinematically change their gait for an intent change to be registered. That is, only the kinematic changes are measured and not the change in *desired* speed, which is a precursor to the kinematic changes. This could be problematic for individuals, perhaps with a spinal cord injury, who cannot actuate their own limbs. Previous work has shown, however, that the motor current draw measurements from powered exoskeletons are indicative of the user’s intentions even when those intentions are not significant in the kinematic signals (Gambon et al., 2019). This means that it is still promising that an intent change could be identified for these individuals with this algorithm as long as at least some of the sensors are related to the internal kinetics of the human/robot system.

## 5. Conclusions

The proposed walking speed intent identification algorithm represents a data-efficient approach that was successful at identifying subjects’ intended change in walking speed in less than one gait cycle for all but the smallest deviations in walking speed. The accuracy and responsiveness of the algorithm depend on the type and the size of the intended speed change. The best results can be expected for SU intent changes of larger magnitudes. Modeling the user’s constant-speed sensor readings with a series of Gaussian distributions enabled the use of the MD as a useful tool to account for the expected variation in each measurement signal. The key to the model’s data efficiency was the simplifying assumption that each of these distributions was independent in time. The constant-speed gait model converges with just 5 min of training data and is still able to reach an accuracy of 87% for the largest SU changes, enabling fast, user-specific model training appropriate for time-restrictive applications (e.g., physical therapy settings).

## Data Availability

Data related to this study will be made available on IEEE DataPort upon acceptance of this article.
